# Low and High-Intensity Ultrasound in Dairy Products: Applications and Effects on Physicochemical and Microbiological Quality

**DOI:** 10.3390/foods9111688

**Published:** 2020-11-18

**Authors:** América Chávez-Martínez, Raúl Alberto Reyes-Villagrana, Ana Luisa Rentería-Monterrubio, Rogelio Sánchez-Vega, Juan Manuel Tirado-Gallegos, Norma Angélica Bolivar-Jacobo

**Affiliations:** 1 Facultad de Zootecnia y Ecología, Universidad Autónoma de Chihuahua, Periférico Fco. R, Almada km 1, Chihuahua C.P. 31453, Mexico; arenteria@uach.mx (A.L.R.-M.); rsanchezv@uach.mx (R.S.-V.); jtirado@uach.mx (J.M.T.-G.); p199385@uach.mx (N.A.B.-J.); 2 Catedrático CONACYT, Av. Insurgentes Sur 1582, Col. Crédito Constructor, Alcaldía Benito Juárez, Mexico City C.P. 03940, Mexico

**Keywords:** dairy products, ultrasound, low-intensity, high-intensity

## Abstract

Milk and dairy products have a major role in human nutrition, as they contribute essential nutrients for child development. The nutritional properties of dairy products are maintained despite applying traditional processing techniques. Nowadays, so-called emerging technologies have also been implemented for food manufacture and preservation purposes. Low- and high-intensity ultrasounds are among these technologies. Low-intensity ultrasounds have been used to determine, analyze and characterize the physical characteristics of foods, while high-intensity ultrasounds are applied to accelerate particular biological, physical and chemical processes during food product handling and transformation. The objective of this review is to explain the phenomenology of ultrasounds and to detail the differences between low and high-intensity ultrasounds, as well as to present the advantages and disadvantages of each one in terms of the processing, quality and preservation of milk and dairy products. Additionally, it reviews the rheological, physicochemical and microbiological applications in dairy products, such as raw milk, cream, yogurt, butter, ice cream and cheese. Finally, it explains some methodologies for the generation of emulsions, homogenates, crystallization, etc. Currently, low and high-intensity ultrasounds are an active field of study, and they might be promising tools in the dairy industry.

## 1. Introduction

The modern food industry is committed to delivering constantly higher-quality, safer and environmentally friendly products. Ultrasonication is a resourceful and feasible technique recognized for its low processing costs and ecological nature with a wide range of applications [1]. In the dairy industry, ultrasonication is being used to improve homogenization [2], enzyme inactivation [3], fermentation [4,5,6,7], nutrient composition [8,9,10,11,12,13], rheological properties [14,15], microbial inactivation [10,12,16,17,18,19,20], the reconstitution of milk powder [21], reduction of the allergenicity of cow’s milk proteins [22] and production of paraprobiotics and postbiotics [8].

An ultrasound (US) has a frequency range within 20 Hz and 1 GHz, which are above the human hearing threshold [23]. For the food industry, two types of ultrasounds might be useful: high- and low-intensity ultrasounds. This classification is based on the ultrasound waves intensity: high-intensity ultrasounds (HIU) use ultrasound intensities >3 W/cm^2^ and low-intensity ultrasounds (LIU) <3 W/cm^2^. However, they can also be classified as (i) high-power low-frequency (20–100 kHz), (ii) medium-power medium-frequency (100 kHz–1 MHz) and (iii) low-power high-frequency (1–100 MHz) [14]. LIU is a nondestructive technique; meanwhile, HIU modify the physical and chemical properties of foods; hence, they might be useful when processing dairy products such as yogurt [14,24,25,26,27], fermented beverages [6,28], cheese [18], milk [15], prebiotic beverages [17], whipped cream [29], whey protein [9,30], butter [31], ice cream [27] and dairy beverages [11]. HIU damage cell structures [32]; therefore, they are also suitable for microbial inactivation in dairy products [19,33] and other liquid foods, such as fruit and vegetable juices [4,17,33]. US combined with heat are called thermosonications, but if pressure is also added, then they are known as manothermosonications [3,6]. US can be applied either directly or indirectly [6]. The direct mode is approximately 100 times more powerful than the indirect one, as the energy flows from the transducer to the sample. However, the direct application has a higher risk of contamination, since the probe is submerged directly in the liquid, while the indirect technique is carried out in baths [34].

## 2. Phenomenology of Ultrasound Waves

This section will explain acoustic waves, frequency spectrum, ultrasound, differences between low- and high-intensity US and non-Newtonian fluids (which is how milk behaves).

### 2.1. Acoustic Waves

Acoustic waves—also known as mechanical or elastic waves—require a propagation medium in order to drive and spread. Acoustic waves propagate through fluids, gases, solids and biological tissues. The linear acoustic wave equation is represented by the equation

(1) $$\nabla^{2}P\left(r,t\right)-\frac{1}{c^{2}}\frac{\partial^{2}P\left(r,t\right)}{\partial t^{2}}=0,$$

where $\nabla^{2}$ is the three-dimensional Laplacian operator, which can be applied to different geometries; this will depend on the type of wave being analyzed, which can be plane, cylindrical and/or spherical waves. *P* is the acoustic pressure of the wave. r is the position vector of the wave and is given by $r=x\hat{i}+y\hat{j}+x\hat{k}$, in Cartesian coordinates. $\hat{i},\;\hat{j}$ and $\hat{k}$ are the unit vectors. *c* is the longitudinal acoustic phase speed of the propagation medium. *t* is the time. $\partial^{2}$ represent the second partial derivative of the function; in this case, the pressure is position- and time-dependent. Equation (1) represents a homogeneous wave equation, which means that it does not matter what or who generates the acoustic waves.

The solution of the harmonic plane wave equation is represented by the equation

(2) $$P=Ae^{-i\left(k\cdot r\;-\;\omega t\right)}+Be^{i\left(k\cdot r\;-\;\omega t\right)},$$

where *A* and *B* are constants that can be determined by applying initial and/or boundary conditions, ***k*** is the wave propagation vector and is given by $k=k_{x}\hat{i}+k_{y}\hat{j}+k_{z}\hat{k}$, *i* is the imaginary number and *k* is the wave number and is defined as $k=\frac{\omega }{c}$. *ω* is the angular frequency. Additionally, *c* can be represented as

(3) c= λ × f,

where λ is the wavelength and *f* is the frequency. Finally, λ can also be represented as

(4) $$\lambda =\;\frac{2\pi }{k}$$

**Figure 1:**
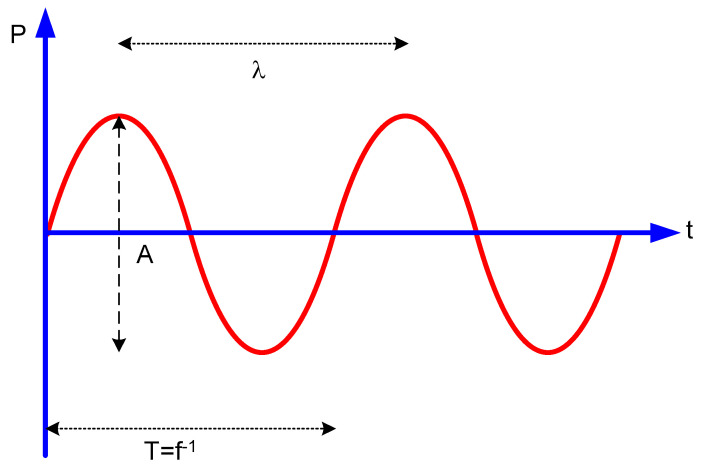
Acoustic wave parameters. P is the pressure wave (Pascal). A is the amplitude; the highest point of the wave is the crest, and the lowest is the valley. λ is the wavelength (meter), T is the period (second) and f is the frequency (Hertz).

The acoustic wave is described by the following parameters: amplitude, wavelength, frequency and period (Figure 1).

**Figure 2:**
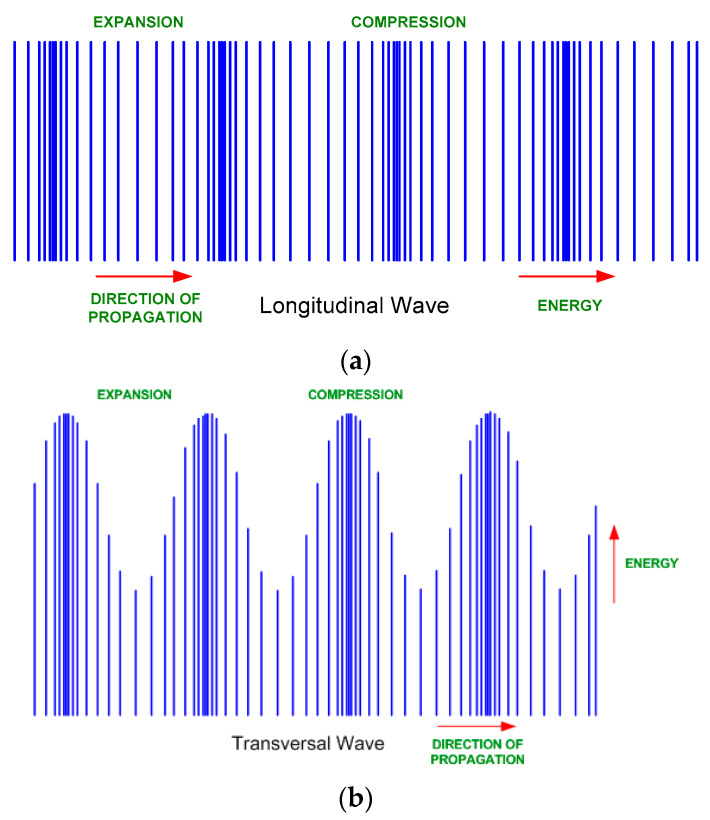
Acoustic waves. (**a**) Longitudinal acoustic wave. (**b**) Transverse acoustic wave.

There are longitudinal and transverse acoustic waves. In the longitudinal acoustic waves, the acoustic energy travels in the same direction of propagation of the wave; on the contrary, the energy of the transverse acoustic waves travels perpendicular to the propagation of the wave. Figure 2 shows the longitudinal and the transverse acoustic waves [35].

**Figure 3:**
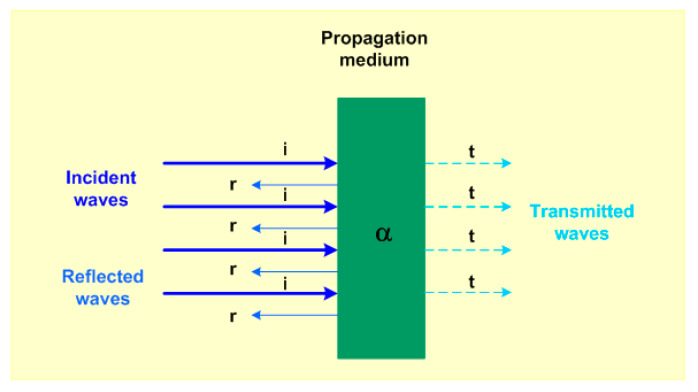
With normal incidences to the surface of the propagation medium, the acoustic waves are incident, reflected and transmitted. α is the attenuation that exists when the incident plane wave passes through a propagation medium.

In this context, acoustic waves also comply with Snell’s law optics [36], where the incident and reflected and transmitted waves are involved in a propagation medium where $\frac{sin\theta_{i}}{c_{i}}=\frac{sin\theta_{t}}{c_{t}}$, $\theta_{i}$, $\theta_{t}$, $c_{i}$ and $c_{t}$ represent the angle of the incident wave, transmitted wave, acoustic longitudinal propagation speed of the incident and transmitted wave, respectively. The attenuation phenomenon is the loss of energy given the intrinsic properties of the propagation medium, the frequency and thickness and is represented by the equation

(5) $$P=P_{o}e^{-i\alpha r},$$

where $P_{o}$ is the amplitude of the initial pressure; *i* is the imaginary number; α is the attenuation coefficient of the medium and losses due to absorption, diffraction, dispersion and scattering. The object of the study is also included. ***r*** is the position vector of the acoustic wave, as shown in Figure 3.

From Figure 3, the following equation can be described:

(6) $$P_{i}={\tilde{P}}_{i}e^{i\left(k\cdot r\;-\;\omega t\right)},$$

(7) $$P_{r}={\tilde{P}}_{r}e^{-i\left(k\cdot r\;+\;\omega t\right)},$$

(8) $$P_{t}={\tilde{P}}_{t}e^{i\left(k\cdot r\;-\;\omega t\right)},$$

where *P_i_*, *P_r_* and *P_t_*, represent the incident, reflected and transmitted pressure waves. ${\tilde{P}}_{i}$ represents a constant that can be determined by applying the initial conditions of the process and/or the environment.

Finally, all the materials have an important acoustic property called the specific acoustic impedance (Rayls), which is given by the product of the volumetric density *ρ* (kg/m^3^) and the acoustic phase propagation speed *c* (m/s); this is Z=ρ × c. The specific acoustic impedance is the resistance that a material presents to the flow propagation of the acoustic wave. It should be clarified that, in solid materials, there are longitudinal waves and transverse waves, so the speed of propagation of the longitudinal and transverse acoustic phase propagations is taken into account [37].

On the other hand, one of the main parameters that distinguishes acoustic waves is the frequency spectrum. Table 1 describes the working frequency spectrum of acoustic waves.

Depending on the frequency range used, it is the application that is being developed; for example, infrasound is an acoustic wave below the range of audible sound and has applications in animal training and biomedical instruments [38]. The audible sound is the range we use to communicate (hearing/speaking); the applications are diverse, such as in communications, musical instruments, etc. Ultrasound is a range that has applications in industries such as nondestructive tests, as well as in the health area, where it has a wide range of applications in biomedical imaging and lithotripsy [39]. Finally, the hypersound is a frequency range where the acoustic wave is applied in the study of devices such as acoustic metamaterials [40] and surface sensors [41]. In this review, we will focus on the ultrasound frequency spectrum, where there are two types of intensities: low and high, which have different functions.

### 2.2. Ultrasounds

Ultrasounds are acoustic waves that travel through a propagating medium within a frequency range between 16 kHz and 1 GHz. This is classified as LIU and HIU. LIU is useful for exploring and characterizing the acoustic properties of materials, while HIU modifies the biological, physical and chemical properties of materials. In most cases, both LIU and HIU ultrasounds use ultrasonic sensors, which have a piezoelectric material as the central operating element.

### 2.3. Piezoelectric Materials

**Figure 4:**
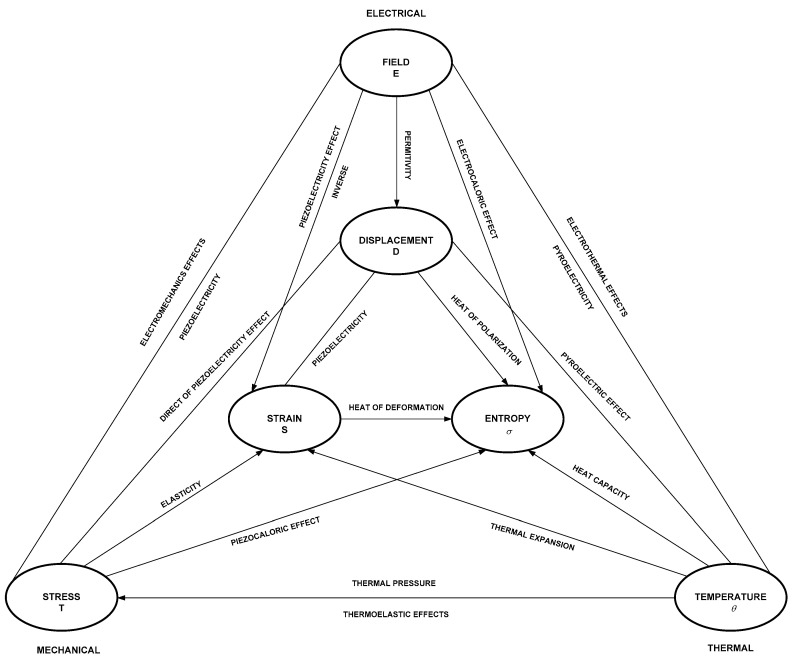
Heckmann’s piezoelectricity diagram [45].

Piezoelectric materials generate electrical pulses when subjected to mechanical stress and generate mechanical stress when applying electrical pulses (Figure 4). There are natural piezoelectric materials such as quartz, piezopolymers like polyvinylidene difluoride (PVDF), piezoceramics like lead zirconate titanate (PZT) and piezocomposites like lead scandiumniobate-lead magnesium niobate-poly(2,6)-naphthalene naphthalate-lead strontium zirconium titanate (PSMNZT) [42,43]. A HIU emitter has a backing, a pair of piezoelectric ceramics in a sandwich configuration and a horn, whose function is a mechanical amplifier. This type of configuration of ultrasonic emitters is called Langevin transducers [44].

### 2.4. Low-Intensity Ultrasounds

**Figure 5:**
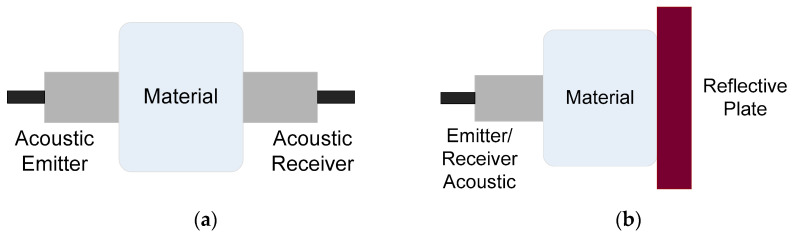
Determination of the acoustic properties of materials through the configurations: (**a**) transmission and (**b**) pulse echo.

LIU determine and characterize the acoustic properties of materials through nondestructive tests. This methodology is known as ultrasonic spectroscopy, which has various configurations; the most common are transmission and pulse echo (Figure 5).

Transmission ultrasonic spectroscopy consists of determining the time it takes for the acoustic wave to pass through the material. This configuration has two ultrasonic sensors: one is the emitter and the other is the receiver, and the sample is placed between both sensors. The emitter sends an acoustic wave that passes through the sample, and the sensor detects the acoustic wave from where the frequency or time spectrum is obtained. The thickness of the sample, the attenuation and, consequently, the acoustic phase velocity can be measured; other mechanical (rheological) properties can be assessed, depending on the signal processing.

Pulse-echo ultrasonic spectroscopy also determines the time it takes to send, pass through and to reflect an acoustic wave sent by a single ultrasonic sensor, which is configured as an emitter/receiver. The sample is placed between the ultrasonic sensor and a reflector plate (the plate is optional). The ultrasonic spectrum obtained (signal processing) is the spectrum related to the phase changes during the experiment.

Both configurations use ultrasonic gel to match the acoustic impedances on the boundaries between the sensors and the sample or the fluid where the sample and sensors are embedded in order to reduce noise and signal losses. The acoustic characterization of materials can be done inside an acoustic chamber as well, where water is the propagation medium and impedance coupler.

### 2.5. High-Intensity Ultrasounds

**Figure 6:**
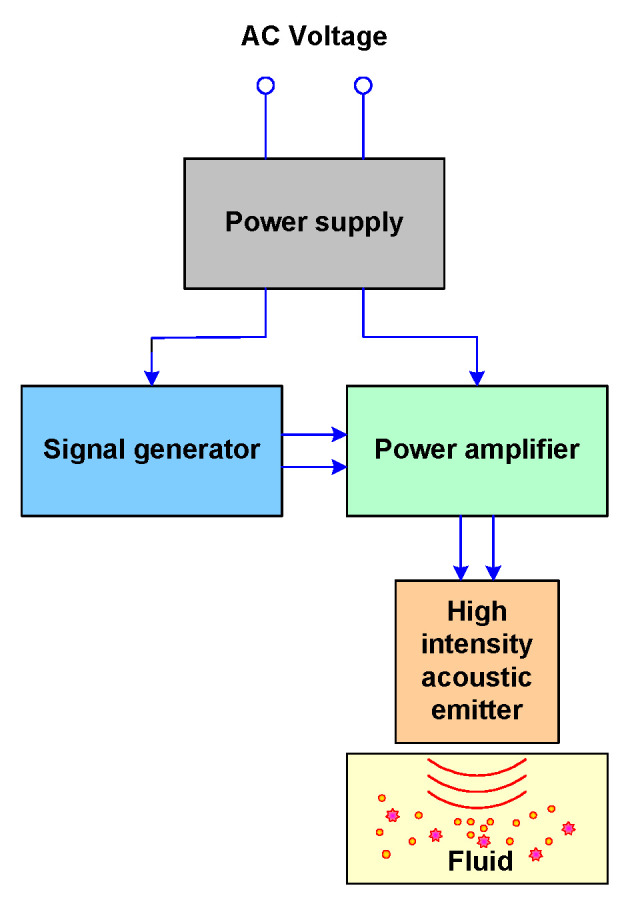
Stages to generate a High-Intensity ultrasound.

HIU modify the biological, physical and chemical properties of organic and inorganic materials through destructive tests. The power supply, signal generator, power amplifier and high-intensity acoustic emitter are the blocks that generate HIU, as shown in Figure 6.

The power supply is a device that powers the signal generator and the power amplifier. This is basically an interface between the alternating current voltage (VAC) power line and the signal generator and power amplifier. The power supply rectifies filters and stabilizes the alternating current signal into a direct current signal, thereby operating the rest of the blocks. The signal generator provides a signal—typically sinusoidal—and sends it to the power generator. This amplifies the signal and increases it to the power and sends the acoustic emitter of high intensity, which generates very strong vibrations and, consequently, generates the effect of acoustic cavitation.

### 2.6. Acoustic Cavitation

**Figure 7:**
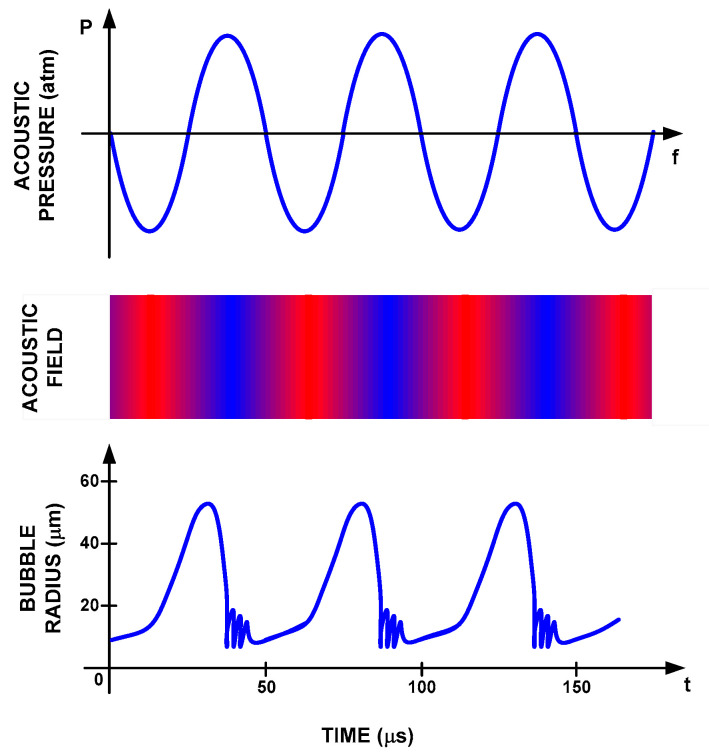
Formation and collapse of bubbles in a transitory state.

High-intensity ultrasounds, under steady-state and/or transient conditions, generate the phenomenon known as acoustic cavitation—that is, the generation, growth and collapse of bubbles. This happens in the transition from the negative half-cycle to the positive half-cycle (expansion and compression) of the acoustic wave. In the case of steady, the number of bubbles increased, and, in stochastic situations for the transitory state, the bubbles implode, as well as the acoustic microjets and the shock waves [46], as shown in Figure 7.

There is a wide field of study on this phenomenon, where it has been discussed in which science acoustic cavitation originated and its description, as well as the denomination of the new areas of study, which are known as sonoluminescence, sonophysics or sonochemistry [47,48,49]. In addition, other areas have been added, depending on the application, such as mechanochemistry [50].

**Figure 8:**
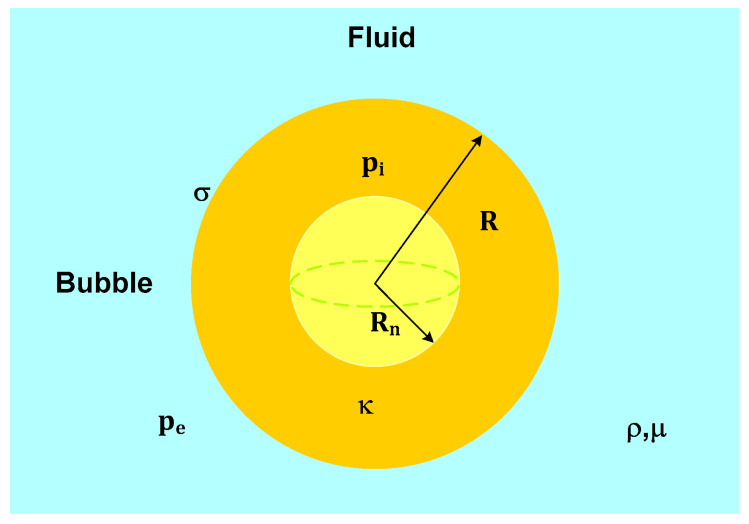
Physical parameters of the bubble in the Rayleigh model. *R* is the radius of the bubble. *R_n_* is the radius of the sphere at rest. *p_i_* is the pressure inside the bubble. *p_e_* is the external pressure of the fluid. *κ* is the polytropic exponent of the gas inside the bubble. *ρ* is the bulk density. *µ* is the dynamic viscosity. *σ* is the surface tension of the fluid.

There are various physical-mathematical models that describe acoustic cavitation thoroughly, such as the Rayleigh model (Figure 8). These models represent the phenomenon of a single bubble under ideal conditions [51] (Figure 8). The model describes a spherical bubble embedded in a fluid, where *R* is the radius of the bubble dependent on time—that is, *R(t)*. *R_n_* is the radius of the sphere at rest. *p_e_* is the external pressure of the fluid. *p_i_* is the pressure inside the bubble.κ is the polytropic exponent of the gas inside the bubble. *ρ* is the bulk density. *µ* is the dynamic viscosity, and σ is the surface tension of the fluid.

This phenomenology is represented by Equation (9), where the points on *R* (one and two points) mean the first and second derivatives with respect to time (Newton’s notation), respectively.

(9) $$\rho R\ddot{R}+\frac{3}{2}\rho {\dot{R}}^{2}=p_{i}-p_{e},$$

Then, the Rayleigh-Plesset model [52] was generated, given by Equation (10):

(10) $$\rho R\ddot{R}+\frac{3}{2}\rho {\dot{R}}^{2}=p_{gn}{\left(\frac{R_{n}}{R}\right)}^{3\kappa }+p_{v}-p_{stat}-\frac{2\sigma }{R}-\frac{4\mu }{R}\dot{R}-p\left(t\right),$$

with

(11) $$p_{gn}=\frac{2\sigma }{R}+p_{stat}-p_{v},$$

and

(12) $$p\left(t\right)=-p_{a}sin\left(2\pi v_{a}t\right),$$

where *p_gn_* is the gas pressure inside the bubble. *p_stat_* is the static pressure, and *p_v_* is the vapor pressure. *p(t)* is the external pressure applied to the wall of the bubble. *υ_a_* is the frequency, and *p_a_* is the pressure amplitude.

Later, the Gilmore model [53] was developed, represented by Equation (13):

(13) $$\left(1-\frac{\dot{R}}{C}\right)R\ddot{R}+\frac{3}{2}\left(1-\frac{\dot{R}}{3C}\right){\dot{R}}^{2}=\left(1+\frac{\dot{R}}{C}\right)H+\frac{\dot{R}}{C}\left(1-\frac{\dot{R}}{C}\right)R\frac{dH}{dR},$$

with

(14) $$H=\int_{{p|}_{r\to \infty }}^{{p|}_{r\;=\;R}}\frac{dp\left(\rho \right)}{\rho },$$

(15) $$p\left(\rho \right)=A{\left(\frac{\rho }{\rho_{0}}\right)}^{n_{T}}-B,$$

(16) $${p|}_{r=R}=\left(p_{stat}+\frac{2\sigma }{R_{n}}\right){\left(\frac{R_{n}^{3}\;-\;bR_{n}^{3}}{R^{3}\;-\;bR_{n}^{3}}\right)}^{\kappa }-\frac{2\sigma }{R}-\frac{4\mu }{R}\dot{R},$$

(17) $${p|}_{r\to \infty }=p_{stat}+p\left(t\right),$$

(18) $$C=\sqrt{c_{0}^{2}+\left(n_{T}-1\right)H},$$

where *C* is the acoustic velocity near the wall of the bubble. *c*_0_ is the acoustic velocity under normal conditions. *H* is the enthalpy. *A*, *B* and *n_T_* are the Van der Waals parameters.

Finally, the Keller-Miksis model [54] describes the behavior of a bubble under quasi-realistic conditions, given by Equation (19) [53]:

(19) $$\left(1-\frac{\dot{R}}{C}\right)R\ddot{R}+\frac{3}{2}{\dot{R}}^{2}\left(1-\frac{\dot{R}}{3C}\right)=\left(1+\frac{\dot{R}}{C}\right)\frac{p_{l}}{\rho }+\frac{R}{\rho c}\frac{dp_{l}}{dt},$$

with

(20) $$p_{l}=\left(p_{stat}+\frac{2\sigma }{R_{n}}\right)\left(\frac{R_{n}}{R}\right)3\kappa -p_{stat}-\frac{2\sigma }{R}-\frac{4\mu }{R}\dot{R}-p\left(t\right),$$

(21) $$p\left(t\right)=p_{a}sin\left(2\pi v_{a}t\right).$$

**Figure 9:**
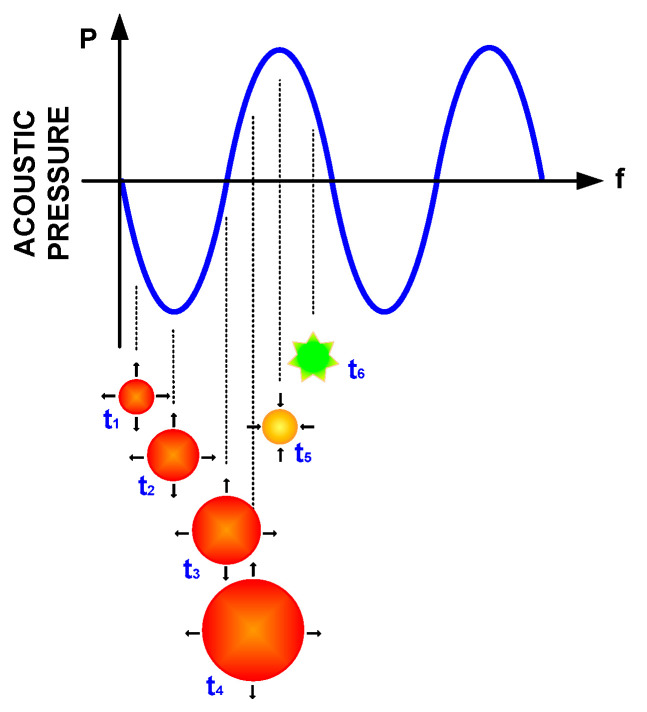
Bubble generation, transition, growth and collapse.

Acoustic cavitation in steady-state conditions generates bubbles and the division between them, given the oscillations in the acoustic field. On the other hand, acoustic cavitation in a transient state refers to the variation of pressure in the short periods with respect to time and the breaking of the molecular bonds of the fluid, rapidly increasing the temperature [55]. If the time-dependent pressure wave is discretized, it is assumed as time t_i_ of growth in the negative half-cycle and the collapse of the bubble in the positive half-cycle, as shown in Figure 9.

If a fluid (liquid) is under shear stress, the bubbles within the fluid increase in size. Hence, bubbles are generated within the fluid. The cavities with vapor increase their size until reaching a maximum volume—therefore, the wave when changing from peak to valley in pressure; the potential energy obtained during growth is transformed into kinetic energy in the implosion. The cavities collapse to sizes even smaller than the originally generated bubbles [56].

The implosions of the bubbles neighboring the surface of an interface are asymmetric and result in microjets of the fluid that hits on the surface of the interface. The average speed of the microjets is of the order of 100–340 m/s and is dependent on the pressure profile and the initial diameter of the bubbles, ranging from 10 to 100µm [57].

There are commonly two types of configurations in the generation and accumulation of bubbles in an acoustic field: the formation of a bubble cloud and the formation of bubble filaments.

The multi-bubble acoustic cavitation formation can be represented by the Keller-Miksis model, as is shown in the following equation [58]:

(22) $$\begin{array}{c} \left(1-M_{i}\right)R_{i}{\ddot{R}}_{i}+\frac{3}{2}\left(1-\frac{M_{i}}{3}\right){\dot{R}}_{i}^{2}=\left(1+M_{i}\right)\frac{1}{\rho_{li}}\left[\rho_{li}-p_{\infty }-p_{si}\left(t+t_{R_{i}}\right)\right]+\frac{t_{R_{i}}}{\rho_{li}}{\dot{\rho }}_{li}-\\ \underset{j}{\sum }\frac{{\left(2R_{j}{\dot{R}}_{j}^{2}+R_{j}^{2}{\ddot{R}}_{j}\right)|}_{t-\frac{r_{ij}}{c_{l\infty }}}}{r_{ij}}, \end{array}$$

where *R_i_(t)* is the radius of the ith bubble. *ρ_li_* is the volumetric density of the fluid outside the ith bubble. *p_∞_* is the ambient pressure. $p_{si}\left(t\right)=p_{a_{i}}sin\left(\omega t\right)$ is the modulated acoustic pressure of the *i*th bubble. tRi≡Ricli, *c_li_* is the acoustic velocity of the fluid outside the ith bubble. $p_{li}=p_{gi}\left(R_{i},t\right)-\frac{4\eta R_{i}}{R_{i}}-\frac{2\sigma }{R_{i}}$ is the pressure on the fluid side of the *i*th wall of the bubble. This model represents the interaction of the *i*th bubble with the rest of the set of bubbles.

### 2.7. High-Intensity Ultrasound Equipment

**Figure 10:**
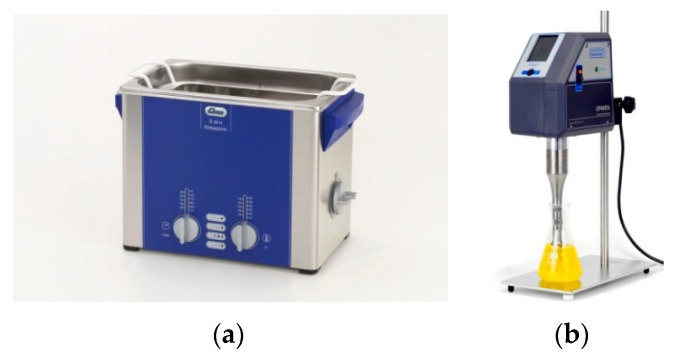
High-intensity ultrasound equipment. (**a**) Ultrasonic bath. (**b**) High-intensity ultrasound system, sonotrode type.

The equipment most used in research are the ultrasonic baths and the ultrasonic emitters or sonotrodes, since these are easily operated (see Figure 10).

## 3. Ultrasounds Applied to Dairy Products

**Figure 11:**
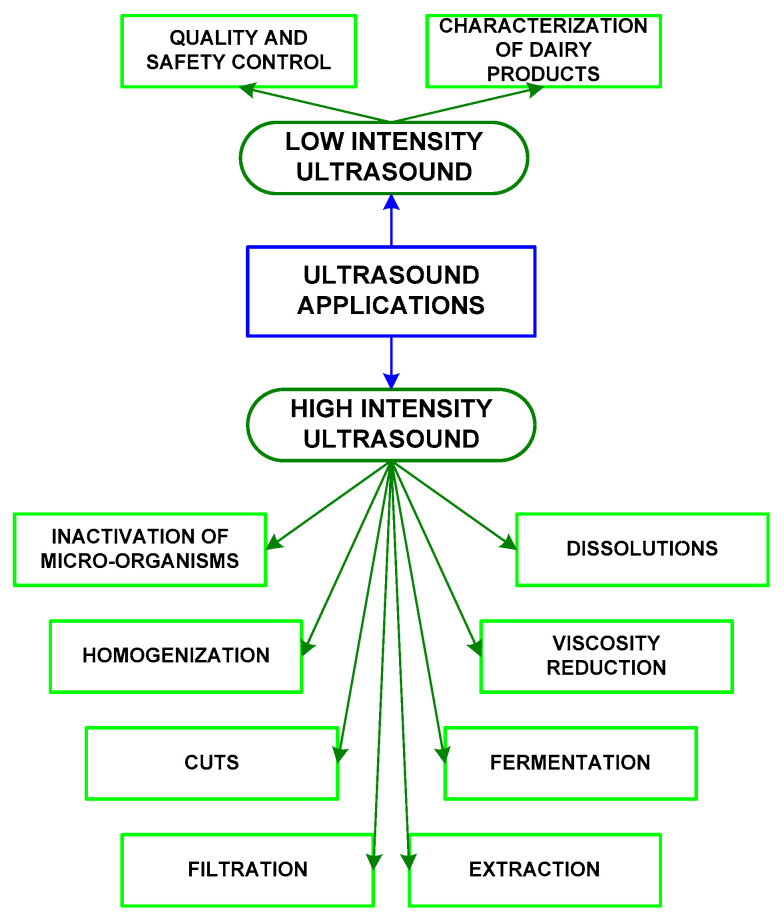
Applications of low-intensity and high-intensity ultrasounds.

Ultrasounds are a nonthermal technology that determines the acoustic properties of foods, and it might modify its properties (see Figure 11).

**Figure 12:**
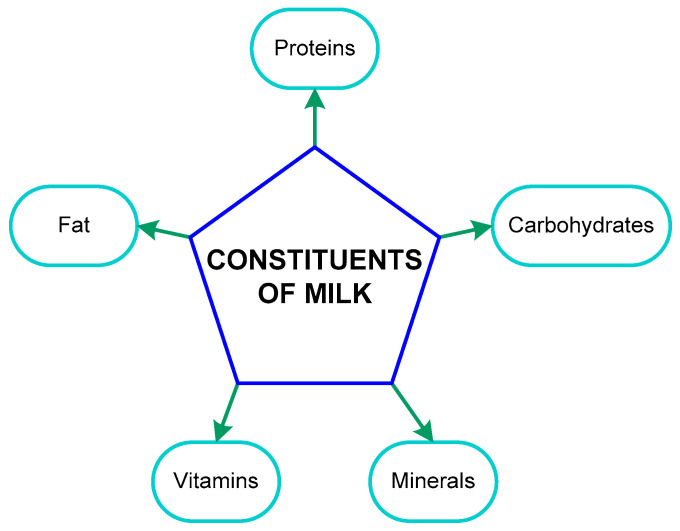
Constituents of milk.

Milk is a basic and nutritious food ingredient in many countries worldwide. The intrinsic properties of milk and dairy products have a central role in child development and growth (see Figure 12).

**Figure 13:**
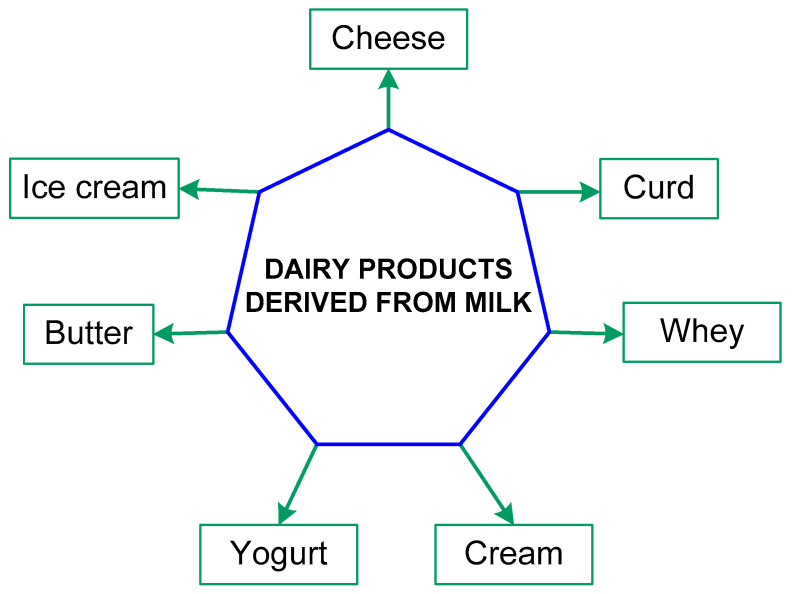
Dairy products derived from milk.

Milk behaves like a non-Newtonian fluid—that is, a fluid whose viscosity varies with the shear stress and the temperature applied to it [59]. For this reason, and with different treatments, various milk derivatives can be generated, as shown in Figure 13. As an emerging technology, US have been applied to a variety of dairy products to improve their quality.

### 3.1. Low-Intensity Ultrasoundsin Dairy Production

Low-intensity ultrasounds do not have a destructive effect on the material component, and their applications are more diversified in medicine. However, in the food industry, their applications are focused as a quality control technique. LIU have been applied to monitor microbial growth, enzymatic reactions, control of the fermentation process, gelling processes and rennet coagulation properties of milk [60,61], as well as they have been used in different stages of cheese and yogurt elaboration. Likewise, in cheese production, they are used to determine the texture, maturity, quality, rheological properties and cutting times during the process [62,63,64,65,66].

Most of the studies that relate the texture and rheological properties with the acoustic properties have been carried out on cheeses, coagulation processes and fermentations. Milk coagulation is the base for the development of several dairy products; hence, it is important to assess and describe each stage under different conditions, such as rennet- and acid-induced milk coagulation and heat-induced gelation of whey proteins [67]. The previous conditions demonstrated that, in rennet-induced milk coagulation, there are three stages: in stage one (pH > 5.1, without changes in the rheological parameters),there is no aggregation or gel formation, during stage two (4.85 < pH < 5.1, G′′ frequency rises and the pH decreases), aggregation starts and, in stage three (pH < 4.85, G′ and G′′ increase quickly with pH, which demonstrates the formation of a three-dimensional gel network of casein particles in milk), the gel is formed in its totality [62,67]. Likewise, LIU have been used to determine coagulation times, which, in the cheese industry, determines the final quality of the product [68].

Benedito et al. [69] reported that LIU could be applied to monitor cheese maturation. They measured the ultrasonic velocity in Mahon cheese with a couple of narrow-band ultrasonic transducers (1 MHz, 0.75-in crystal diameter), a pulser-receiver and a digital storage oscilloscope coupled to a personal computer. Ultrasonic velocity increased with the ripening time from 1630 to 1740 m/s, for soft and hard cheeses, respectively. This behavior was a consequence of the changes in the elastic properties of the cheese during maturation (increase in the modulus of deformation) due to moisture losses. The parameters of the texture profile analysis (TPA) were correlated with the ultrasonic velocity during the maturation of Mahon cheese, and the deformation modulus presented a higher correlation. The ultrasonic velocity in solid materials depends on the material density and the elastic modulus, the latter having the most influence. Benedito et al. [66] applied LIU to evaluate the ripening of Manchego cheese between 29 and 296 days at different temperatures (4, 8, 12 and 17 °C). The internal and external textures of Manchego cheese increased during maturation. Compression tests presented a higher correlation with ultrasonic; the best correlations were in compression work (*R*^2^ = 0.843) and hardness (*R*^2^ = 0.826). In this type of cheese (high porosity), the ultrasonic waves propagation was limited by the holes present; the majority of them were scattered. There was no observed relationship between the temperature coefficient of the ultrasonic velocity and the moisture content. However, under all the temperatures at which the tests were performed, the ultrasonic velocity increased with the ripening, as previously reported by Benedito et al. [69] in Mahon cheese.

Fermented dairy products are made from the acid coagulation of milk, normally caused by lactic acid bacteria (LAB). During this process, the caseins micelles reduce their charge and precipitate. It has been observed that ultrasonic velocity and attenuation changes depend nearly completely on milk serum composition changes and that casein micelles have little contribution to the release of calcium [70].

In terms of yogurt manufacture, LIU is useful when following the fermentation process [71]. Microbial growth can induce variations in the ultrasonic propagation parameters in packed milk; hence, US are a prominent noninvasive tool to evaluate microbial contamination in milks [72].

Of all the fermented dairy products, yogurt is the most representative of them, and viscosity is one of the attributes that most influences its quality and sensory acceptance [26]. Rheological parameter (storage (G′) and elasticity (G″) modulus) evaluations are conducted through viscometers and rheometers, which are considered destructive techniques [73,74,75]. In this sense, Izbaim et al. [73] characterized the yogurt fermentation process by means of ultrasound (5 MHz), relating the ultrasonic velocity with the modulus G′ of the medium. The results showed that the time-of-flight decreased during milk fermentation, which meant an increase in the ultrasonic velocity. According to the authors, the ultrasonic velocity is directly related to the elastic modulus of the system. By comparing the curves of the amplitude and time of flight, the time of flight indicated four phases in the evolution of the amplitude during milk fermentation. In the third phase, the flight time reached a plateau to later decrease moderately and remain almost constant (fourth phase). The third phase was identified around 125 min after the fermentation started, suggesting that, at this point, the fermentation should be stopped.

Low-intensity ultrasounds have been used as a nondestructive technique to monitor the coagulation and gel formation process in milk protein suspensions, reconstituted milk and fresh milk [76,77]. During their formation, gels present a very short deformation region in which their viscoelastic parameters remain constant. For this reason, the monitoring of their formation, with rheological techniques, must be carried out at very small strains, since external forces break their structures [75]. In this sense, AT-cut quartz crystals have been used as sensors at very low strains in the analysis of biomolecular interactions in liquid solutions. Other authors [74] observed the gel formation process in milk added with Glucono-δ-lactone (GDL) by means of AT-cut quartz crystals and calculated the G′ and G″ modulus from the imaginary parts of the impedance, R and X, respectively. The behavior of viscoelastic parameters G′ and G″ and the tangent loss (δ) in the gel layer in contact with the quartz cell were similar to those reported by other authors who used rheological techniques to evaluate the same process.

Dwyer et al. [78] evaluated the microstructural changes in milk during the renneting process using high-resolution ultrasonic spectroscopy in combination with dynamic rheology. The process over time was monitored by the evolution of ultrasonic velocity and attenuation at a frequency of 2 to 15 MHz. The authors reported that the behavior of velocity and ultrasonic attenuation during the renneting process was described by the scattering of the ultrasonic waves on gel aggregates. The results obtained by the ultrasonic technique were in agreement with those obtained by oscillating rheological methods. However, the results showed that the ultrasonic technique was more sensitive during the rennet coagulation process and can be described since pre-gelling. These results were similar to those reported by Wang et al. [79], who applied ultrasonic (7.8 MHz) and oscillating rheological methods to evaluate the rennet gel formation of a whey protein-free casein solution preheated to an ultra-high temperature (UHT). The normalized ultrasonic attenuation as a function of time presented a behavior similar to the modulus G′; both parameters increased during the coagulation process. The coagulation times obtained by both methods were linearly correlated (*R*^2^ = 0.9967); however, the highest values were obtained by oscillatory rheological measurements. These results suggest that the ultrasonic technique was more sensitive and detected protein aggregation earlier than the rheological method. Koc and Ozer [80] developed an ultrasonic technique to evaluate the rennet coagulation of whole milk for cheese production. To determine the coagulation time, the authors calculated the ultrasonic attenuation coefficient from measuring the ultrasonic wave amplitude and time of flight for a known distance. The results evidenced a linear correlation (*r*^2^ = 0.88) between the coagulation start times estimated by ultrasonic attenuation and viscosity measurements. All the results described above demonstrated that nondestructive techniques based on low-intensity ultrasounds present results similar to those obtained by rheological methods, and, in some cases, the sensitivity of ultrasonic techniques is superior.

### 3.2. High-Intensity Ultrasoundsin Dairy Production

HIU modify the biological, physical and chemical properties of organic and inorganic materials through destructive tests, and they have been used during the production of various dairy products [1,6,8,33,81,82,83,84,85,86,87,88,89]. The effectiveness of HIU in milk and dairy products is widely documented, and it depends on frequency, power and amplitude of the ultrasound used and time applied [29].

#### 3.2.1. Fermentation Process

The fermentation is a biochemical process, which involves some microorganisms known as starter cultures; they transform complex organic compounds into simpler compounds [90]. Fermentation requires the most resources and time in the dairy industry; therefore, alternatives are sought to reduce fermentation times and costs and shorten production stages [6,8].

Parameters such as intensity, frequency, composition of the food (matrix), type of equipment (probe/bath) and duration influence the reduction in fermentation times achieved with low-frequency (intensity lower than 1 W/cm^2^) ultrasounds [6]. Furthermore, the reduction is related to the microbial metabolism of carbohydrates and organic acids [8]. Ultrasounds alter or damage the cell releasing the enzyme β-galactosidase; thus, the concentration of glucose and galactose increases, making them more available for bacteria [6,28].When fermentations are carried out, certain factors must be considered to conduct an efficient fermentation process, such as the power and frequencies of ultrasounds, processing time, type and duration of the pulses and growth phase [7].

It has been mentioned that, for fermentation to take place, the phenomenon of cavitation must not occur [8]; however, cavitation might favor the affinity of the enzyme for the substrate therefore, the hydrolysis reaction is increased [4]. The formation of pores in the bacterial cell membrane increases the permeability, enzyme production and facilitates mass transfer, accelerating the exchange of the nutrient and bacterial growth rate and metabolism in starter cultures [90].

In yogurt production, fermentation encompasses the time between the addition of the starter culture and the moment in which the pH reaches 4.7 [6,67]. If the fermentation time decreases, some characteristics such as texture and consistency can be improved [6]. The fermentation time can be reduced using US with a frequency of 20 kHz in the presence of *Lactobacillus delbrueckii* [91]; the same was observed using the milk of Jersey and Holstein cows [6].

Flow cytometry analyses have shown that 15 s of US application (amplitude of 60%, time 15 s and 10-g/L peptone, 50-mL samples) are sufficient to change the membranes of *Lactobacillus casei* subsp. *Casei* (ATTC 39392).These conditions improved the lactic acid production and increased the biomass production [4].

Likewise, using low-frequency US (22 ± 1.65 kHz, 1-L samples) to reconstitute powdered milk yields a higher nutritional final product, with an increment in the concentration of bioactive compounds. This treatment, when applied prior to fermentation, improves the dispersion of the final product, which favors the conditions in which the bacteria carry out this process [13].

Meanwhile, in fermented dairy products, the viscosity and texture are quality attributes valued by the consumer; these characteristics depend to a great extent on the microstructure of the gel [6,14,26,92,93]. In general, the gel should have a smooth and creamy texture, with a low syneresis level [26,92,94]. The application of US in the manufacture of these types of products affects their rheological and textural properties in a desirable or undesirable way, which depends on the stage of the process where the US is applied. Riener et al. [94] applied thermosonication (24 kHz, 400 W, 90 °C, 10 min, 200-mL sample) in milk to obtain yogurt. The results showed that, in comparison with the yogurt obtained from conventionally heated milk (90 °C for 10 min), the US promoted an increase of up to 25% in the G′ modulus, thus evidencing more rigid gels. Later, the same authors [92], under the same conditions described above, reported a similar rheological behavior in yogurt. In addition, the authors evaluated the yogurt texture by applying a texture profile analysis (TPA). Compared with the yogurt obtained from conventionally heated milk, the thermosonications promoted a 100% increase in yogurt hardness (1.8 N) and a significant increase in chewiness and gumminess as result of a more compact structure in the gel network. According to several authors, cavitation during thermosonication breaks casein micelles and fat globules; therefore, caseins interact with partially denatured whey proteins, and these complexes stabilize the new interfaces generated by the breakdown of fat globules. These phenomena promote a more compact network that results in gels with higher values in their viscosity, modulus G′ and hardness without modifying their non-Newtonian behaviors of the pseudoplastic type [14,92,93]. Gursoy et al. [14] compared the effect of thermosonication (24 kHz, 70 °C, 15 min, 800-mL sample) at different ultrasound powers (100, 125 and 150 W) and the conventional heating (10 min 90 °C) in milk for yogurt production. All samples showed non-Newtonian pseudoplastic behavior (shear-thinning), whose apparent viscosity decreases with the increasing shear rate. Viscosity decreased with the increasing potency; this behavior is desirable in the production of yogurt drinks.

Thermosonication as a treatment previous to fermentation that offers the possibility of obtaining gels with rheological properties superior to those offered in those obtained from conventionally heated milk. However, HIU have also been applied during the fermentation process, and the results have not been the most desired for this product. Nöbel et al. [26] applied HIU (45 kHz, power density of 17 kW/m^3^) during the fermentation of skim milk. The ultrasound treatment resulted in the formation of lumps and increased graininess, which are textural defects in yogurt. The pH of yogurts played an important role in the formation of these particles; pH of 5.4 and 5.1 were defined as critical values. At these pH´s, the system has a low zeta potential (ζ), so the collision between particles under these conditions results in a large number of molecular interactions that form large gel aggregates.

These results were similar to those reported by Körzendörfer et al. [25], who evaluated the effect of the US (35 kHz, 300 W, 5 min, 100-mL sample) yogurt fermentation process (pH 5.2, 42 °C). The gel firmness, measured through a penetration test, was lower in the ultrasonic samples (109–157 mN), compared to the samples without US treatment (152–217 mN). The ultrasonic samples were granulated and with a large presence of larger gel particles. The results reported by Nöbel et al. [74] and Körzendörfer et al. [25] were in contrast to the results observed by Körzendörfer et al. [95]. These authors applied HIU (20 kHz, 20 W, 43.5 °C) during fermentation (pH 5.8 to 5.1) to produce Greek yogurt with a protein content of 10%. Compared to the control (yogurt without US treatment), ultrasonication reduced the number of particles from 316 to 225, and the size particles decreased from 1.37 to 1.28 μm. The authors suggest that the power under which the US was applied disturbs fermentation; however, the shear forces generated by cavitation break the large gel particles again. Regarding the rheological properties, the modulus G′ decreased with sonication, from 2489 Pa in the control sample to 644 Pa in the sonicated sample. A similar behavior was observed in the apparent viscosity (η100), which decreased from 2.53 to 1.52 Pa∙s for the control and sonicated samples, respectively. According to the authors, the rheological properties developed by sonication during the fermentation of Greek yogurt could facilitate the subsequent stirring within the production process of this yogurt type.

#### 3.2.2. Microbial Inactivation

Ultrasounds are a mild-preservation technology, and they might be considered as an alternative or reinforcement to antimicrobial heat treatments in foods and dairy products. Ultrasounds can inactivate pathogens and enzymes, which increase the microbial shelf-life, without affecting the starter cultures significantly [8,96,97].

Ultrasounds cause an acoustic phenomenon that promotes bubble formation and their instant destruction [12,98,99]. The imploding bubbles emit shockwaves that lead to the formation of eddies. The areas where the eddies form are zones of intense stress and energy that, eventually, fracture and rupture the cell wall [12,99,100]. Hence, the inactivation effect is caused by the mechanical damage of the cell wall and the membranes [98], the inactivation starts rapidly causing extensive cell injury and, then, it diminishes gradually [96,100]. The intensity of the effect is related with the frequency; for example, low-frequency ultrasounds were lethal for *Enterobacter aerogenes*, but the same effect was not observed with high frequencies [101].

The inactivation effect depends on several factors: (1) eddie size. Eddies larger than the cells that push or move them instead of shear them; hence, smaller eddies are more disruptive [99,102]. (2) Intrinsic factors: morphology, size and cell wall. The morphology of the bacterial cell influences its resistance to sonication. In general, it is considered that cocci, as *Staphylococcus aureus*, are more resistant than rod-shaped bacteria; this may be due to the size and surface area. Cocci are smaller with less surface areas, rendering them more resistant to ultrasonication [103]. In terms of size, larger cells tend to be more sensitive than smaller ones. Large cells have more surface areas and might be bigger than the eddies, as mentioned previously [103]. The cell walls vary among microorganisms; they are thinner and more complex in Gram-negative bacteria than in Gram-positive. Gram-positive bacteria are more resistant to sonication than Gram-negative [104], and this resistance is linked to the peptidoglycan network [99]. It is important to mention that the phase of the growth curve also modifies the effect of the ultrasound due to changes in the cell wall; for instance, in the period between the exponential and the stationary phases, the peptidoglycan wall changes significantly. Additionally, slow-growing bacteria or bacteria in the stationary phase have a denser and thicker peptidoglycan layer [99]. (3) Processing conditions, mainly due to the hurdle effect; for example, microbial reductions are higher in psychrophiles sonicated at higher temperatures [103,105]. Finally, (4) the sample volume; larger volumes form larger eddies and reduce the number of eddies per unit, which reduces the disruption efficiency [106,107].

Apart from the factors above, milk and dairy products exert a sonoprotective effect on bacteria such as *Listeria monocytogenes*, *Escherichia coli* and *Pseudomonas fluorescens* [98,101]. Complex systems, such as those created by the addition of inulin, whey, lactose and other sugars, hinder the transfer of energy produced by the acoustic cavitation through the beverage—thus, protecting the bacteria [17,98,108]. The antimicrobial effect can be deemed selective, as starter cultures are not as sensitive as other bacteria commonly found in milk, mainly because most of the starter cultures are Gram-positive [99,109].

On another hand, high-power ultrasounds can be coupled with heat (thermosonication). The main advantage of this procedure is the pasteurization with temperatures lower than those used in the conventional method. In this regard, Monteiro et al. [12] applied thermosonication processing (19 kHz, 400 W) at different energy densities (0.3–3.0 kJ/cm^3^) as a nonthermal alternative to high-temperature short-time pasteurization (HTST, 72 °C/15 s) to pasteurize chocolate-flavored milk. The results showed that, with the increase in energy density, a product with a higher flow index (*n*) was obtained but with a lower consistency index (*k*). Compared with conventional pasteurization, the ultrasounds decreased the size of the fat globules at the same time that they denatured the proteins; this change in the microstructure of the product promoted a more Newtonian behavior.

#### 3.2.3. Foam

In some cases, foams are undesirable in industrial processes due to problems with process control or equipment handling. In this sense, an ultrasonic processing (20 kHz at 20 °C during three min) removed foam (up to 80%) and dissolved oxygen in reconstituted skim milk [110]. Riera et al. [111] proposed the ultrasound as an excellent alternative to foam minimization.

#### 3.2.4. Homogenization

**Figure 14:**
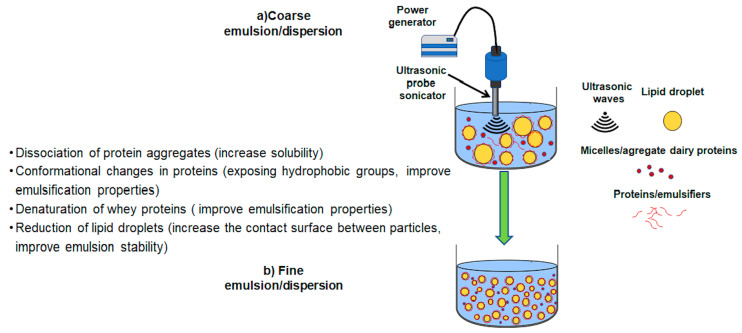
Schematic representation of ultrasonic homogenization. (**a**). Coarse emulsion/dispersion. (**b**). Fine emulsion/dispersion.

Figure 14 shows the homogenization process, which is applied to homogenize the oil droplet size in coarse emulsions and improve the solubility of dairy ingredients in aqueous mediums (Figure 14a). During homogenization with ultrasounds, ultrasonic waves give rise to the phenomenon of cavitation, which generates shear forces that deform and break large, fat droplets into smaller droplets, increasing the interfacial area [112,113]. Amphiphilic substances present in the medium, such as proteins, are adsorbed into the new oil-water interfaces to stabilize the newly formed droplets. In the case of milk proteins, ultrasonic cavitation promotes a dissociation of protein micelles and a reduction in the size of the protein aggregates, improving their solubility [113,114]. Ultrasonic cavitation improves the emulsifying properties of milk proteins due to conformational changes and their denaturation [113]. Finally, the obtained product will present a smaller particle size distribution than the original (Figure 14b).

Homogenization by ultrasound takes place as a consequence to this technology to mix some immiscible liquids without the use of additives [115,116]. Homogenization by ultrasound has been applied to baby food, ketchup, milk products, dressings, fruit juice and mayonnaise, among others [115].Ultrasounds can be supplied through bath or horn systems, but there is another device called “liquid whistle”, which was developed for homogenization [116]. This device generates vibrations via the flow of a liquid. The operating conditions, such as pressure or throughput, are determined by the use of sized orifices varying the velocity until reaching the particle size or degree of dispersion required [116]. Skim milk (60 mL) processed by ultrasound at 20 kHz for up to 60 min showed a reduction in the globule fat size until around 10 nm [117]. In this context, Crudo et al. (2014) reported an effective homogenization of milk fat particle sizes up to nanoscale dimensions after ultrasonication treatment (35 kHz at 30–37 °C) [118]. Bermúdez-Aguirre et al., 2008 evaluated the influence of thermosonication treatment (24 kHz, 400 W, 120-μm amplitude at 63 °C for 30 min) on the microstructure of fat globules in whole milk. Milk fat globules exhibited disintegration as a result of ultrasonication, reducing their sizes until <1 μm, favoring the amalgamation of casein and serum proteins [119].

A comparative study between the ultrasonic homogenization of milk (20 kHz during 5 and 10 min at 55 °C and conventional homogenization (high pressure of 200 bar at 55 °C) demonstrated that the size range of fat globules in ultrasonically homogenized milk samples was much smaller (0.725 µm) than conventional (2–5 µm). However, the mean size and size distribution of the fat globules were dependent on the ultrasonic power and duration of sonication [120].

#### 3.2.5. Emulsification

**Figure 15:**
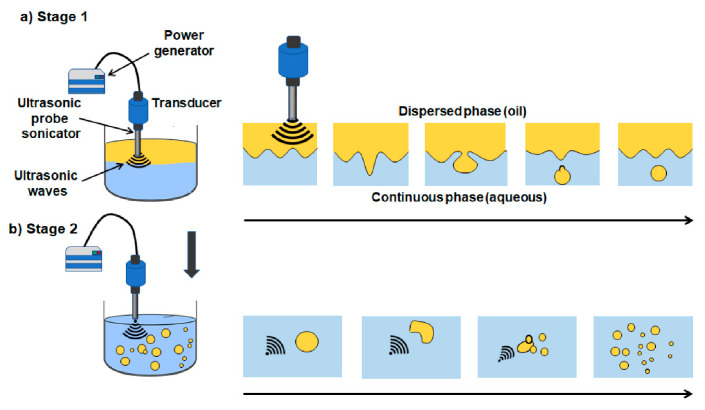
Schematic representation of ultrasonic emulsification. (**a**) The shear forces generated during acoustic cavitation near the interface between the aqueous phase and oil phase promote the eruption of large oil drops (dispersed phase) in the continuous aqueous phase. (**b**) The oil droplets formed in the first stage are reduced to smaller droplets as a consequence of the shock waves generated during cavitation (adapted from Perdih et al., 2019 and Plüisch and Wittemann, 2016).

Figure 15 shows the emulsification process by applying a high-intensity ultrasound. According to Perdih et al., 2019 and Plüisch and Wittemann, 2016 [121,122], starting from two immiscible phases, the shear forces generated during acoustic cavitation near the interface between the aqueous phase and oil phase promote the eruption of large oil drops (dispersed phase) in the continuous aqueous phase (Figure 15a). Subsequently, the oil droplets formed in the first stage are reduced to smaller droplets as a consequence of the shock waves generated during cavitation. Under the influence of the ultrasonic waves, the breaking and coalescence of the fat droplets promote a more stable state with a narrower droplet dispersion (fine emulsion, Figure 15b). Furthermore, in dairy products, the new droplets formed are stabilized by the presence of emulsifier substances such as milk proteins.

High-density ultrasounds have also been evaluated in emulsification processes; the shear forces caused by cavitation destroy the fat droplets, thus reducing the particle size distribution and, consequently, increasing the interfacial area. In this line, Aslan and Dogan [112] reported that ultrasounds can improve the stability of emulsions based on dairy ingredients. These authors applied US (24 kHz for 3 min, 85 W/cm^2^) to obtain emulsions from olive oil (7%, 10% and 15%); skim milk (11%); sucrose (14%); xanthan (0.2%) and monodiglycerides (0.2%). Compared with the emulsion obtained by mechanical stirring (control), the emulsions obtained by the US showed lower viscosity and a reduction in the consistency index (*k*). According to the authors, the shear forces generated by cavitation broke the oil droplets into smaller particles and allowed a better distribution of the proteins on the new interfaces in the system, thus reducing the viscosity. The ultrasound treatment for emulsion production decreased the droplet size and creaming index and increased the zeta potential (ζ).

#### 3.2.6. Crystallization

**Figure 16:**
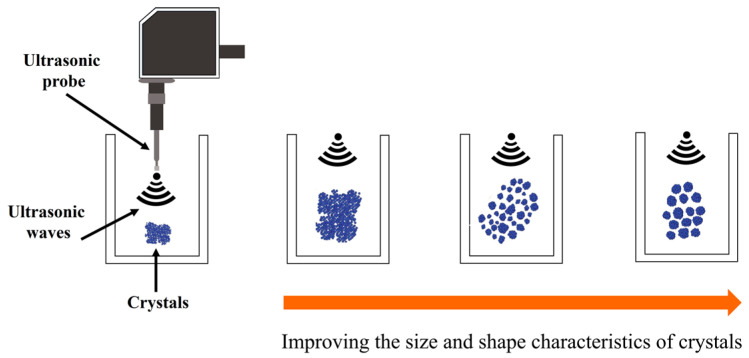
Schematic representation of the sonocrystallization process.

Ultrasounds could affect the crystallization. Before crystals can form, there must exist in the solution a number of solid bodies (nuclei), referred to as the centers of crystallization. Nucleation can occur spontaneously or by the influence of some external stimulus (agitation or mechanical shock, friction, among others) [123]. Crystallization occurs as a consequence of freezing, where a uniform size distribution of crystals is in the function of diverse factors such as fluctuations in temperatures and pressures and ineffective cooling, among others. Acoustic cavitation can promote nucleation in a phenomenon referred to as sonocrystallization (Figure 16) [91,124], which is also defined as the use of power ultrasounds to control the crystallization process, commonly used during the nucleation phase of crystallization [125].

In the case of ice cream, crystals are formed during freezing, where the aim is to obtain a product with the smallest crystal size distribution as possible. The application of ultrasounds during ice cream-making has proven extremely useful in crystallization processes, as it can initiate seeding and control the subsequent crystal growth. Likewise, the freezing process is accelerated, which leads to a better quality of the product. This is because US promote crystal fragmentation and, therefore, promote the production of smaller pure crystals of a uniform size. It also prevents crystal fouling on the cold surface and maintains a high rate of heat transfer [124,126,127]. These findings could suggest that ultrasound processing is effective in the preparation of ice cream. Nonetheless, as is well-known, ice cream contains up to 50% by volume of air, and an ultrasound is an efficient degasser, which could alter the texture of the ice cream [91]. It has been suggested that the use of a tube sonicator upstream of the scraped surface exchanger is the ideal approach for ice cream, as well as augments the initial gas content [128].

On the other hand, Martini et al. [129] analyzed the effect of ultrasound processing on the crystallization behavior and microstructure of anhydrous milk fat to improve the physicochemical characteristics and nutritional qualities of lipid-based foods [129]. The authors observed that ultrasounds diminished the induction time of crystallization (faster crystallization) with smaller crystals. The samples showed increased viscosity after sonication due to crystallization.

Bund and Pandit [125] studied the effects of sonication (22 kHz, 10-mL sample) on the rate of the lactose recovery (lactose solution containing 85% (*v/v*) ethanol at pH 4.2) and crystal habit [125]. They reported that 87.45% of lactose was recovered after two min of sonication, with respect to the conventional lactose recovery process, which takes anywhere between 12 and 72 h for only 55–60% recovery. In addition, sonicated samples showed rapid and higher lactose recoveries in comparison to non-sonicated samples in the absence, as well as presence, of protein. These findings were associated with the cavitation-assisted rapid lowering of the vapor pressure of the solution due to the presence of ethanol, which reduced the solubility of lactose in the ethanol/water mixture. It was also reported that ultrasound processing improved the size and shape characteristics of lactose crystals [122].

#### 3.2.7. Milk Proteins

Proteins isolated from milk are commercialized in the form of powders that are subjected to a rehydration process at the time of use; however, protein powder concentrates do not dissolve easily and can form aggregates. On the other hand, applying heat treatments to facilitate the solubilization of dairy ingredients can represent a problem, since the properties of these materials are modified [130,131]. In addition to the physicochemical changes that these substances experience during drying, their hydration properties decrease during storage [132,133]. Therefore, the application of US for the hydration of dairy ingredients was proposed, since the use of high temperatures can be omitted. In this sense, Chandrapala et al. [117] applied ultrasounds (20 kHz, 450 W, 60-mL samples) in suspensions of casein mixed with whey proteins; it was observed that US decreased the viscosity by up to 40%, and this behavior increased with the concentration of whey protein. Ultrasounds broke the whey protein-casein aggregates, without detecting structural changes in these milk components. Another advantage of the application of ultrasounds in these types of materials is the improvement of their stability. In this sense, Ashokkumar et al. [130] applied high-intensity and low-frequency ultrasounds (20 kHz, 5 min, 60-mL samples) in preheated (80 °C, 1 min) whey protein suspensions (4–15%). To evaluate the effectiveness of the treatment, the suspensions were subject to post-heating at 85 °C for 5 min. The viscosity of the preheated samples without US increased with respect to the control (unheated milk), and when post-heating was applied, the viscosity increased again. However, when US were applied in addition to preheating, the viscosity decreased significantly, and post-heating had a minimal effect on this parameter. It should be noted that the low viscosity was maintained even after freeze or spray-drying and then reconstitution in an aqueous medium. According to the authors, the mechanism by which the viscosity remained low in the sonicated samples was unclear. However, this stability was attributed to a merely physical process, a consequence of the different shear forces generated during acoustic cavitation. In a similar study, Zisu et al. [134] applied a high-density ultrasound (20 kHz, 59–60 °C) to reduce the viscosity and its increment with the time (age thickening) in medium-heat skim milk concentrates containing 50–60% solids. An ultrasonic unit (20 kHz, 1 kW, energy density of 4–7 J/mL) was coupled to operate in-line with the evaporator (59–60 °C, 2000 mL min^−1^). After evaporation, the milk was sonicated (40–80 W) again for one min. The viscosity was reduced by 10% and 17%, which was a consequence of the shear forces generated during acoustic cavitation that broke the association between the proteins and polymer chains. In addition, sonication delayed the rapid increase in viscosity due to age thickening. In another study, Krešić et al. [131] evaluated the US (20 kHz, 15 min, ultrasonic power: 43–48 W/cm^2^) on the flow properties of suspensions at 10% (*w/w*) of whey protein isolate (WPI, protein = 97.8%) and whey protein concentrate (WPC, protein = 61.3%). It was observed that US increased the solubility of WPC and WPI. The flow curves of all suspensions were adjusted to the Power Law model (*R*^2^ = 0.989–0.998); they presented Non-Newtonian behavior (*n* < 1), considered as shear-thickening (dilatant), characterized by an increase in the apparent viscosity, with the increase in the shear rate. With respect to the control samples (without sonicating), the consistency index (*k*) increased from 0.036 to 0.152 mPa s^n^ and from 0.041 to 0.186 for the WPC and WPI samples, respectively. According to the authors, the increase in apparent viscosity and the *k*-index were the consequences of a higher exposure of the hydrophilic amino acids of the proteins, allowing the proteins to bind a greater amount of water from the medium. In addition, this behavior was proportional to the protein concentration in the system; in this case, it was the sample with WPI.

#### 3.2.8. Cheese Production

Regarding the application of HIU in the cheese production process, most of the applications have been carried out as thermosonication as an option to conventional pasteurization [135,136,137]. Another advantage of high-intensity ultrasound application in cheese production is the reduction of the setting time and an increase in the firmness of the curd [138,139,140]. Bermúdez-Aguirre and Barbosa-Cánovas [132] applied thermosonication (400 W, 24 kHz, 63 °C, 30 min, 500-mL sample) in milk to make Mexican “fresh cheese”. The results showed that HIU increased the fat content and cheese yield. With respect to the control (milk without sonication), the cheese obtained by HIU presented a more homogeneous microstructure as a result of a reduction in the size of the fat globules and a better distribution of the proteins in the system. The cheese had a softer and more brittle texture, which is a desirable characteristic of this cheese. Zhao et al. [140] observed that ultrasound applications (20 kHz, 800 W, 0–20 min) improved the goat milk coagulation properties. The application of the HIU broke the fat globules into smaller particles and increased the denaturation of the proteins. As a consequence, the coagulum strength, final storage modulus, cohesiveness, water-holding capacity and crosslinking of gels increased. In a more recent study, Almanza-Rubio et al. [135] evaluated the effect of thermosonication (20 kHz, 4–63 °C) in modifying the textural and rheological properties of cream cheese at different power levels (0–100 W) and time (3–30 min).The spreadability and the modulus G′ decreased with the sonication at power levels of ≤50 W and a long time (≤30 min) at 50 °C. They found that ultrasound powers lower than 50 W reduced milk fat globule sizes, increasing their retention in the cheese. However, a power level greater than 50 W increased the size of the fat globules due to coalescence. In addition, there was probably a greater denaturation of the proteins, which altered the membranes of the fat globules. These results suggest that the application of ultrasounds allows improving the yield, textural and rheological properties of cream cheese under specific conditions.

## 4. Conclusions

In the last decade, consumers have become more sophisticated, and their purchasing power gives them the possibility to be more exigent-demanding in their food purchases in terms of quality, flavor, freshness, nutrition and availability without neglecting the price and the care of the environment. This represents a challenge for the food industry. In this sense, ultrasonication cavitation has gained popularity in various fields of quality and the processing of dairy products, where it has been used for nondestructive quality testing, homogenization, modifying milk components, reducing protein allergies, microbial destruction, enzymatic inactivation, improving the fermentation and cheese and yogurts quality and enhancing the crystallization of lactose. The ultrasonic energy required for the cited applications is low and relatively easy to scale up to production, with significant savings in the processing costs. However, more research is still required in the applications that US can have at an industrial level and in continuous processes, so that their use in the mass production of dairy foods is consolidated. Finally, it would be interesting to see what ultrasonication can do in other dairy products, such as dulce de leche.

## Figures and Tables

**Table 1:** Acoustic frequency spectrum.

Name	Range, (Hz)
Infrasound	IS < 16
Audible Sound	16 ≤ AS ≤ 17.8
Ultrasound	17.8 < US < 1 G
Hypersound	HS ≥ 1 G
